# Phosphatase and Tensin Homolog (PTEN) of Japanese Flounder—Its Regulation by miRNA and Role in Autophagy, Apoptosis and Pathogen Infection

**DOI:** 10.3390/ijms21207725

**Published:** 2020-10-19

**Authors:** Wenrui Li, Xiaolu Guan, Li Sun

**Affiliations:** 1 CAS Key Laboratory of Experimental Marine Biology, CAS Center for Ocean Mega-Science, Institute of Oceanology, Chinese Academy of Sciences, 7 Nanhai Road, Qingdao 266071, China; liwenrui@qdio.ac.cn (W.L.); guanxiaolu@qdio.ac.cn (X.G.); 2 Laboratory for Marine Biology and Biotechnology, Qingdao National Laboratory for Marine Science and Technology, 1 Wenhai Road, Qingdao 266237, China; 3 University of Chinese Academy of Sciences, 19 Yuquan Road, Beijing 100049, China

**Keywords:** *Paralichthys olivaceus*, microRNA, phosphatase and tensin homolog, pathogen infection, immune defense, autophagy, apoptosis

## Abstract

MicroRNAs (miRNAs) are small non-coding RNAs with important roles in diverse biological processes including immunity. Japanese flounder (*Paralichthys olivaceus*) is an aquaculture fish species susceptible to the infection of bacterial and viral pathogens including *Edwardsiella tarda*. In a previous study, pol-miR-novel_547, a novel miRNA of flounder with unknown function, was found to be induced by *E. tarda*. In the present study, we investigated the regulation and function of pol-miR-novel_547 and its target gene. We found that pol-miR-novel_547 was regulated differently by *E. tarda* and the viral pathogen megalocytivirus, and pol-miR-novel_547 repressed the expression of PTEN (phosphatase and tensin homolog) of flounder (PoPTEN). PoPTEN is ubiquitously expressed in multiple tissues of flounder and responded to bacterial and viral infections. Interference with PoPTEN expression in flounder cells directly or via pol-miR-novel_547 promoted *E. tarda* invasion. Consistently, in vivo knockdown of PoPTEN enhanced *E. tarda* dissemination in flounder tissues, whereas in vivo overexpression of PoPTEN attenuated *E. tarda* dissemination but facilitated megalocytivirus replication. Further in vitro and in vivo studies showed that PoPTEN affected autophagy activation via the AKT/mTOR pathway and also modulated the process of apoptosis. Together these results reveal for the first time a critical role of fish PTEN and its regulatory miRNA in pathogen infection, autophagy, and apoptosis.

## 1. Introduction

MicroRNAs (miRNAs) are a class of small non-coding RNAs expressed by eukaryotic cells that post-transcriptionally regulate gene expression [1,2,3]. The first discovery of functional miRNA occurred in the early 1990s; since then miRNAs have attracted tremendous attention, and many miRNAs with diverse functions have been reported [4,5]. To date, miRNAs are known as important mediators involved in various biological processes, including cellular development and immunity [6,7,8,9,10,11]. MiRNA regulation is achieved through interaction of the miRNA with the 3′-untranslated region (UTR) of the target gene, resulting in RNA-induced silencing complex (RISC), which leads to degradation or translation blockage of the target mRNA [12,13,14]. In fish, miRNAs involved in immune response induced by bacterial and viral infection have been identified and studied in several species, including Japanese flounder (*Paralichthys olivaceus*), grouper (*Epinephelus coioides*), ayu (*Plecoglossus altivelis*), miiuy croaker (*Miichthys miiuy*), and tongue sole (*Cynoglossus semilaevis*) [15,16,17,18,19,20,21,22,23]. However, the in-depth regulatory mechanisms of fish miRNAs are largely unknown.

In mammals, the phosphatase and tensin homolog (PTEN) gene encodes a tumor-suppressing phosphatase that regulates the cellular activities including autophagy, apoptosis, and cell proliferation through the inhibition of the phosphoinositide 3-kinase (PI3K)/AKT signaling pathway [24,25,26]. The loss or mutation of PTEN causes constitutive activation of the PI3K-induced pathway, which eventually leads to tumor development [27,28,29]. In addition to its classical role as a tumor suppressor, PTEN is also considered an immune regulator involved in inflammatory responses [30,31,32,33,34]. In mammals, it has been demonstrated that miRNAs regulate cell apoptosis, proliferation, and autophagy by targeting PTEN [35,36,37]. In fish, studies on PTEN have been documented mainly in zebrafish, in which it was shown that PTEN played an important role in embryonic development, caudal fin-fold regeneration, tumorigenesis, hematopoiesis, and myelocyte migration [38,39,40,41]. In medaka, PTEN was also shown to be involved in embryo development [42].

Autophagy is an evolutionarily conserved process that eliminates harmful or unwanted cellular components and molecules by delivering them to lysosomes for degradation [43,44,45]. Thus, autophagy plays a critical role in cytoprotection under various conditions of cellular stress such as that caused by nutrient deficiency [46,47,48]. Recent studies indicate that autophagy also functions in immune processes, including antigen presentation, regulation of immune system, controlling of the pro-inflammatory response, and host defense against invading pathogens [49,50,51,52].

Japanese flounder is an important aquaculture species in Asian countries including China. *Edwardsiella tarda*, a Gram-negative bacterium, is a severe fish pathogen that causes high mortality in many farmed fish including flounder [53,54]. In a previous study of high-throughput sequencing of *E. tarda*-infected flounder [18], it was found that *E. tarda* regulated the expression of 96 miRNAs of flounder, one of which is pol-miR-novel_547, a novel miRNA with unknown function. In the present study, we identified PTEN as the target gene of pol-miR-novel_547, and examined the involvement of PTEN and pol-miR-novel_547 in pathogen infection, autophagy, and apoptosis. Our results add new insight into the function of miR-novel_547 and PTEN in the antimicrobial immunity of fish.

## 2. Results

### 2.1. Pol–miR-novel_547 Expression Is Regulated by Pathogen Infection

**Figure 1:**
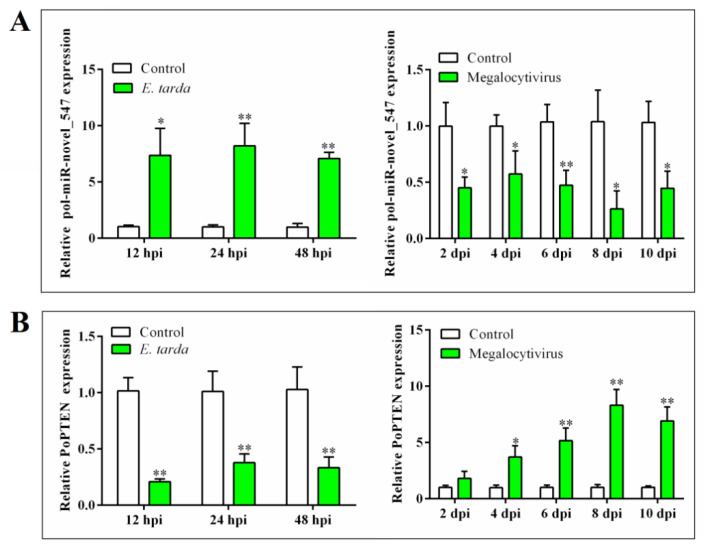
Expression of pol–miR-novel_547 (**A**) and phosphatase and tensin (PTEN) homolog of flounder (PoPTEN) (**B**) in response to bacterial and viral infection. Flounder were infected with or without (control) *Edwardsiella tarda* or megalocytivirus, and the expressions of pol–miR-novel_547 (**A**) and PoPTEN (**B**) in kidney were determined by quantitative real time RT-PCR at different times. hpi, hour post-infection; dpi, day post-infection. Values are the means of triplicate experiments and shown as means ± SD. * *p* < 0.05; ** *p* < 0.01.

qRT-PCR analysis showed that in flounder infected with the bacterial pathogen *E. tarda*, the expression of pol-miR-novel_547 was significantly upregulated at 12, 24, and 48 hpi. In contrast, in flounder infected with megalocytivirus, the expression of pol-miR-novel_547 was significantly downregulated at 2, 4, 6, 8, and 10 dpi (Figure 1A). These results suggest an involvement of pol-miR-novel_547 in pathogen infection.

### 2.2. Pol–miR-novel_547 Negatively Regulates PoPTEN

**Figure 2:**
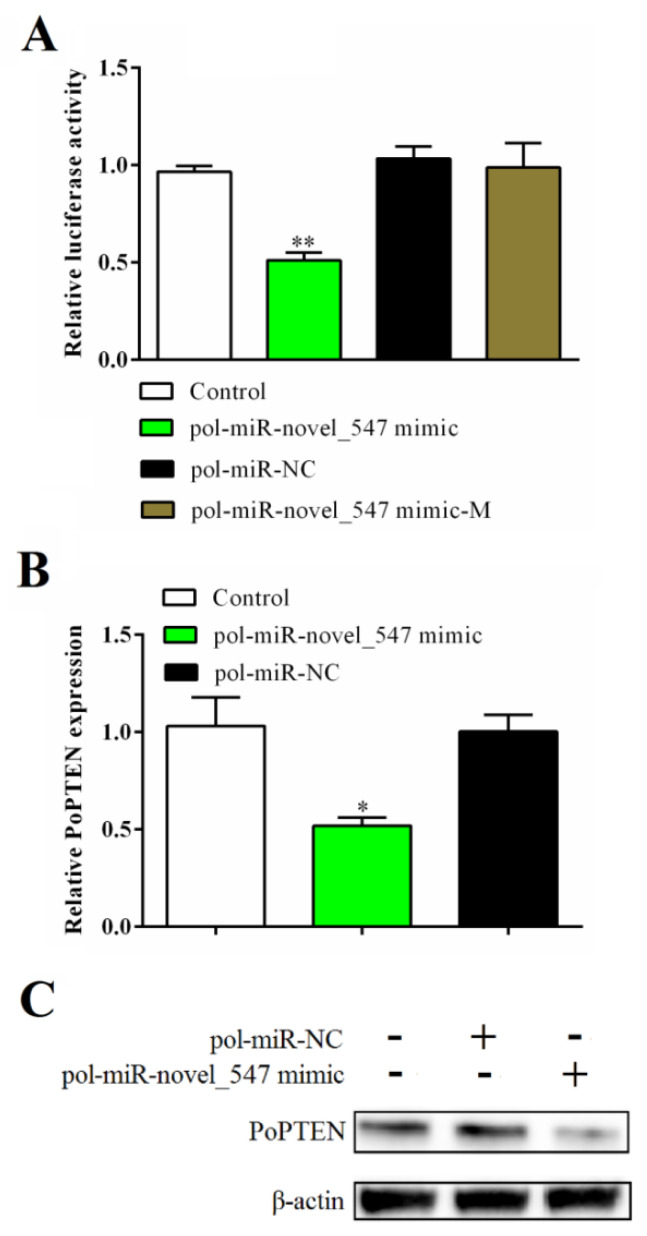
Regulation of PoPTEN expression by pol-miR-novel_547. (**A**) HEK293T cells were transfected with pPTEN-Report in the absence (control) or presence of pol-miR-novel_547 mimic, pol-miR-NC (negative control of pol-miR-novel_547), or pol-miR-novel_547 mimic-M (mutant of pol-miR-novel_547). Relative luciferase activity was determined at 24 h after transfection. (**B**,**C**) FG-9307 cells were transfected with or without (control) pol-miR-novel_547 mimic or pol-miR-NC, and PoPTEN expression was determined by qRT-PCR (**B**) or Western blot (**C**) with β-actin as a loading control. The values of (**A**,**B**) are the means of triplicate experiments and shown as means ± SEM. * *p* < 0.05; ** *p* < 0.01.

Based on sequence analysis, the PTEN gene of flounder (PoPTEN) was predicted to be a target gene of pol-miR-novel_547. To examine whether this was the case, luciferase reporter assay was performed with pPTEN-Report, which carries the 3′-UTR of PoPTEN fused to the luciferase gene. In HEK293T cells co-transfected with pPTEN-Report and pol-miR-novel_547 mimic, the luciferase activity was significantly reduced compared with the control cells (Figure 2A). In contrast, when HEK293T cells were transfected with pPTEN-Report plus pol-miR-novel_547 mimic-M, a mutated form of pol-miR-novel_547, the luciferase activity was not significantly affected (Figure 2A). To examine the effect of pol-miR-novel_547 on the expression of PoPTEN, flounder FG-9307 cells were transfected with pol-miR-novel_547 mimic, and the result showed that the PoPTEN mRNA and protein levels decreased significantly (Figure 2B,C). These results indicate that PoPTEN is a target gene of pol-miR-novel_547 and is negatively regulated by the latter.

### 2.3. PoPTEN Possesses Conserved Structure and Responds to Pathogen Infection

PoPTEN has 422 amino acid residues and contains the conserved tyrosine phosphatase-like catalytic domain of PTEN in the N-terminus (residues 24 to 181) and the C2 domain of PTEN in the C-terminus (residues 188 to 368). It shares 78.89–98.12% overall sequence identities with the PTEN homologs of teleost and mammals (Supplementary Figure S1). In flounder, PoPTEN expression was detected, in the increasing order, in spleen, gill, brain, kidney, blood, intestine, muscle, heart, and liver (Supplementary Figure S2). *E. tarda* and megalocytivirus infections significantly reduced and enhanced, respectively, the expression of PoPTEN in kidney (Figure 1B).

### 2.4. PoPTEN Significantly Affects Bacterial Infection In Vitro and In Vivo

**Figure 3:**
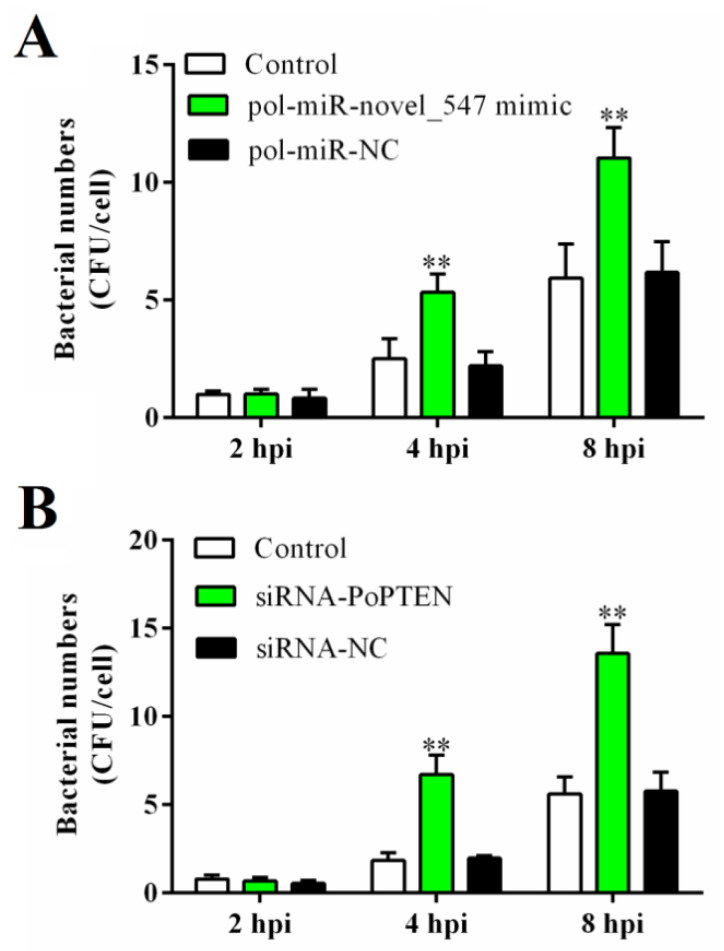
In vitro effects of pol-miR-novel_547 and PoPTEN on bacterial infection in flounder cells. (**A**) FG-9307 cells were transfected with or without (control) pol-miR-novel_547 mimic or pol-miR-NC and then infected with *Edwardsiella tarda*. Intracellular bacterial number was determined at different hours post infection (hpi) and shown as CFU (colony forming unit). (**B**) FG-9307 cells were transfected with or without (control) siRNA-PoPTEN or siRNA-NC and then infected with *E. tarda*. Intracellular bacterial number was determined as above. In both panels, values are the means of triplicate experiments and shown as means ± SEM. ** *p* < 0.01.

**Figure 4:**
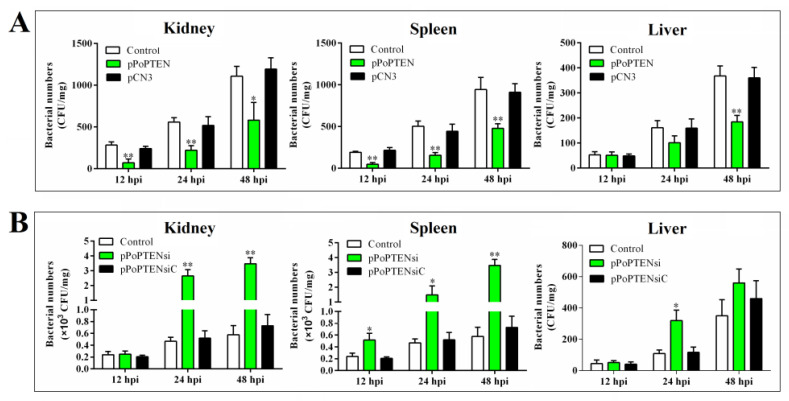
In vivo effects of PoPTEN on bacterial infection in flounder. For PoPTEN overexpression, flounder were treated with or without (control) pPoPTEN or the control plasmid pCN3 (**A**), for PoPTEN knockdown, flounder were treated with or without (control) pPoPTENsi or the control plasmid pPoPTENsiC (**B**). The fish were infected with *Edwardsiella tarda*, and bacterial numbers in kidney, spleen, and liver were determined at different times. Values are the means of triplicate experiments and shown as means ± SD. * *p* < 0.05; ** *p* < 0.01.

To examine the potential effect of PoPTEN on pathogen infection, PoPTEN expression in flounder FG-9307 cells was interfered by transfection with pol-miR-novel_547, or by RNAi mediated via a siRNA (siRNA-PoPTEN), which effectively reduced PoPTEN expression (Supplementary Figure S3). Following *E. tarda* infection, the cells treated with pol-miR-novel_547 or siRNA-PoPTEN exhibited significantly increased bacterial loads at 4 hpi and 8 hpi compared with the control cells (Figure 3). To examine the in vivo effect of PoPTEN, PoPTEN overexpression and knockdown were created in flounder by treating with the plasmids pPoPTEN and pPoPTENsi, respectively, which significantly enhanced and blocked, respectively, the PoPTEN expression (Supplementary Figure S4). *E. tarda* infection showed that in flounder treated with pPoPTEN, the bacterial loads were significantly reduced at 12, 24, and 48 hpi in kidney and spleen, and at 48 hpi in liver (Figure 4A), whereas in fish treated with pPoPTENsi, the bacterial loads were significantly increased in a time-dependent manner (Figure 4B). These results indicate that PoPTEN exerts a significant effect on pathogen infection.

### 2.5. PoPTEN Regulates Autophagy

**Figure 5:**
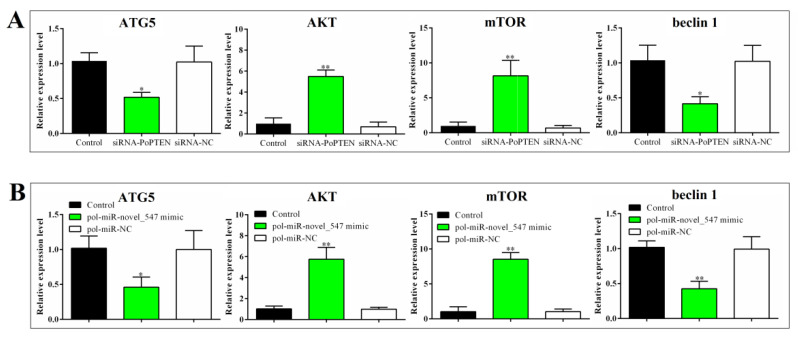
The expression of autophagy-associated genes in flounder cells with PoPTEN knockdown (**A**) and pol-miR-novel_547 overexpression (**B**). (**A**) FG-9307 cells were transfected with or without (control) siRNA-PoPTEN or siRNA-NC for 24 h. (**B**) FG-9307 cells were transfected with or without (control) pol–miR-novel_547 mimic or pol-miR-NC for 24 h. For both panels, the expressions of ATG5, AKT, mTOR, and beclin 1 were determined by qRT-PCR. Values are the means of triplicate experiments and shown as means ± SEM. * *p* < 0.05; ** *p* < 0.01.

**Figure 6:**
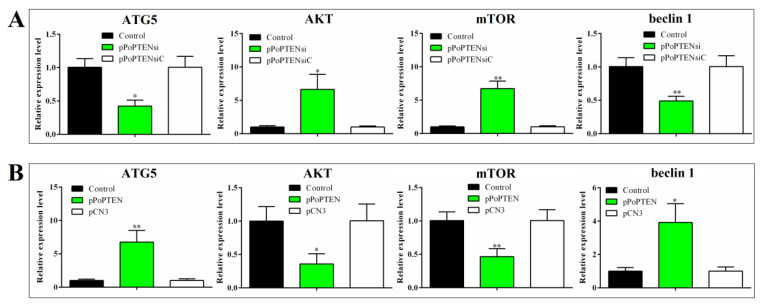
In vivo expression of autophagy-associated genes in flounder with PoPTEN knockdown (**A**) and overexpression (**B**). (**A**) Flounder were administered with or without (control) pPoPTENsi or pPoPTENsiC for 7 days. (**B**) Flounder were administered with or without (control) pPoPTEN or pCN3 for 7 days. For both panels, the expressions of ATG5, AKT, mTOR, and beclin1 in kidney were determined by qRT-PCR. Values are the means of triplicate experiments and shown as means ± SD. * *p* < 0.05; ** *p* < 0.01.

**Figure 7:**
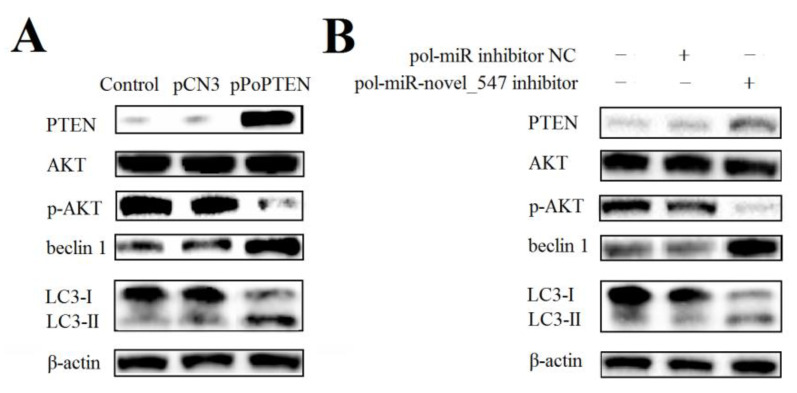
Effects of PoPTEN and pol-miR-novel_547 on the expression of autophagy-related genes in mammalian and fish cells. (**A**) HEK293T cells were transfected with or without (control) pPoPTEN or pCN3 for 24 h. (**B**) FG-9307 cells were transfected with or without (control) pol-miR-novel_547 inhibitor or pol-miR-NC inhibitor for 24 h. For both panels, PTEN, AKT, p-AKT (phosphorylated AKT), beclin 1, and LC3 proteins were detected by Western blot. β-actin was used as a loading control.

qRT-PCR analysis showed that in flounder FG-9307 cells with PoPTEN knockdown induced by siRNA-PoPTEN or pol–miR-novel_547 overexpression, the expressions of the autophagy related genes ATG5 and beclin 1 were significantly decreased, while two other autophagy related genes, AKT and mTOR, were significantly increased (Figure 5). Consistently, in vivo study showed that PoPTEN knockdown in flounder significantly reduced ATG5 and beclin 1 expression but increased AKT and mTOR expression (Figure 6A). In contrast, PoPTEN overexpression in flounder significantly increased ATG5 and beclin 1 expression but decreased AKT and mTOR expression (Figure 6B). When expressed in HEK293T cells, PoPTEN increased the level of beclin 1 protein, induced conversion of LC3-I to LC3-II, and reduced the level of phosphorylated AKT (p-AKT) (Figure 7A). Similar results were obtained in flounder FG-9307 cells transfected with pol-miR-novel_547 (Figure 7B).

### 2.6. Pol-miR-novel_547 Is Involved in Apoptosis

**Figure 8:**
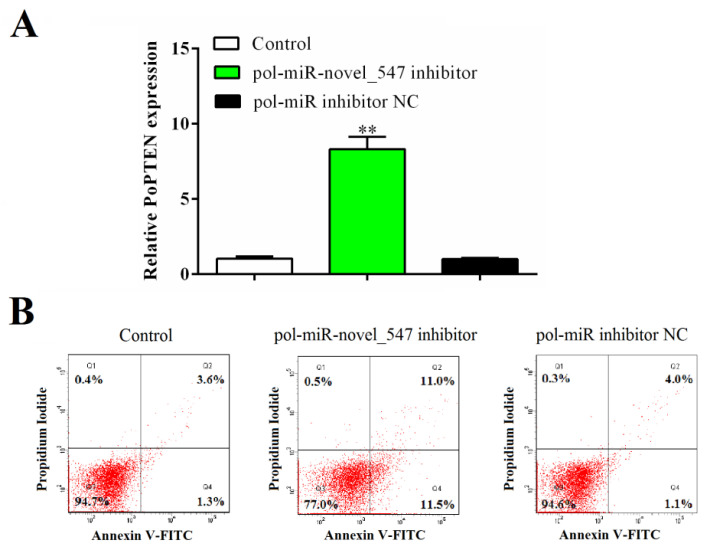
The effect of PoPTEN on apoptosis. FG-9307 cells were transfected with or without (control) pol-miR-novel_547 inhibitor or pol-miR inhibitor NC for 24 h. (**A**) PoPTEN expression in the cells was determined by qRT-PCR. Values are the means of triplicate experiments and shown as means ± SEM. **, *p* < 0.01. (**B**) The transfected cells were labeled with annexin V-FITC and propidium iodide, and apoptosis was determined by flow cytometry.

Flow cytometry analysis showed that in flounder FG-9307 cells with pol-miR-novel_547 inhibition by transfection of pol-miR-novel_547 inhibitor, which significantly enhanced PoPTEN expression (Figure 8A), the apoptotic cells reached 22.5%. This value was significantly higher than that in the control cells (4.9%) or in the cells transfected with the control RNA of pol-miR-novel_547 inhibitor (5.1%) (Figure 8B). These results indicate that pol-miR-novel_547, via PoPTEN, is involved in apoptosis.

## 3. Discussion

Pol-miR-novel_547 is a novel miRNA identified in a previous micro-transcriptome analysis as a miRNA exhibiting significant expressional changes during *E. tarda* infection [18]. Consistently, in the present study, qRT-PCR analysis showed that pol-miR-novel_547 expression was significantly altered during *E. tarda* as well as megalocytivirus infection, implying a role of pol-miR-novel_547 in bacterial and viral infection. For most miRNAs, they exert their biological functions by binding to the 3′-UTR of the target genes, whereby blocking the expression of the target genes [12,55]. Similarly, pol-miR-novel_547 was found to interact with the 3′-UTR of PoPTEN, resulting in repression of gene expression. Furthermore, elevated pol-miR-novel_547 expression in flounder cells significantly reduced the mRNA and protein levels of PoPTEN. These results demonstrated that PoPTEN is indeed a target gene of pol-miR-novel_547 and is inhibited by pol-miR-novel_547.

In mammals, PTEN is a dual protein–lipid phosphatase involved in many cell processes [24,56,57,58]. Studies have shown that the PI3K/PTEN/Akt/mammalian target of rapamycin (mTOR) signaling pathway transmits proliferative signals from membrane-bound receptors to modulate cell growth and apoptosis [59,60]. In this pathway, PTEN dephosphorylates the secondary messenger produced by PI3K and interrupts the downstream activation of AKT [61]. Suppression of the miR-221/222 cluster in gastric cancer cells inhibited cellular proliferation and induced apoptosis by up-regulating PTEN expression [35]. Another miRNA, miR-21, could decrease autophagy by down-regulating PTEN in mesangial cells [37]. In fish, it has been reported that zebrafish PTEN deficiency induced the activation of PI3K/mTOR pathway and AKT, which affected embryo and tissue development [38,41]; madaka PTEN knockout also upregulated PI3K/AKT signaling, which led to abnormal embryo development [42]. In the present study, we found that compared with human and mouse PTEN, fish PTEN homologs are highly conserved in sequence and structure, and, in flounder, PoPTEN was constitutively expressed in a wide range of tissues. These results suggest an essential role of PTEN in the fundamental physiology of lower and higher vertebrates.

MiRNA-mediated regulation is known to play a vital part in antimicrobial immune responses [62,63,64,65,66]. In our study, pol-miR-novel_547 and PoPTEN expressions were regulated by *E. tarda* and megalocytivirus, suggesting an involvement of pol-miR-novel_547 and PoPTEN in pathogen infection. Consistently, enhancing pol-miR-novel_547 expression or interfering PoPTEN expression in flounder cells significantly augmented *E. tarda* infection, whereas PoPTEN overexpression significantly attenuated *E. tarda* infection. These results indicate a requirement of PoPTEN for effective bacterial elimination, which is in line with the previous report that human PTEN deficiency led to susceptibility of multiple types of cells to infection by Mycoplasma and *Mycobacterium bovis* [34]. In addition to *E. tarda*, megalocytivirus infection was also found to be affected by PoPTEN, which markedly promoted the replication of the virus. Together, these observations suggest a vitalness of PTEN-associated cellular process in bacterial and viral infections.

Autophagy is a complicated cellular event involving a large number of autophagy-related (ATG) proteins and regulators [67,68]. To date, at least 41 ATG proteins have been identified, of which, ATG5 is indispensable for modulating the process of autophagy [69,70]. Beclin 1 is a mammalian ATG6 and functions as a core component of the beclin 1-VPS34 Class III PI3K complex, which serves as a platform for the recruitment of other autophagy proteins during autophagosome biogenesis [71,72,73]. The initiation of autophagy is regulated by Class I and Class III PI3K [73]. Class I PI3K inhibits autophagy indirectly via AKT and mTOR, while Class III PI3K directly promotes autophagy through interaction with beclin 1 [73,74]. Previous studies showed that miRNAs and PTEN are important regulators of autophagy [75,76,77,78,79]. PTEN can inhibit the activation of AKT, causing suppression of the PI3K/AKT/mTOR signaling [76,80,81]. MiRNAs, such as miR-93-5p and miR-21, can regulate autophagy via the pathways involving PTEN [82,83]. In our study, both in vivo and in vitro experiments showed that PoPTEN knockdown attenuated ATG5 and beclin 1 expression and promoted AKT and mTOR expression, whereas PoPTEN overexpression produced the opposite effects. Furthermore, PoPTEN overexpression, either directly or through inhibiting pol-miR-novel_547, markedly increased the production of beclin 1 and LC3 II, the highly specific marker of the autophagosome [84], and decreased the production of phosphorylated AKT. These results indicate a positive role of PoPTEN in the activation of autophagy.

Apoptosis is a type of programmed cell death characterized by cell shrinkage and nuclear collapse [85,86,87]. Apoptosis participates in many biologic processes including antimicrobial immunity. Recently, regulation of apoptosis by miRNAs has been reported [88,89,90,91]. In our study, we found that interference with pol-miR-novel_547, which resulted in enhanced PoPTEN expression, significantly promoted the apoptosis of flounder cells, implying an involvement of pol-miR-novel_547 and PoPTEN in the regulation of apoptosis. This observation is in line with the pro-apoptotic role of PTEN in mammals [36,92,93,94].

In summary, we demonstrated in this study that flounder PTEN is regulated by pol-miR-novel_547 and plays an important part in autophagy, apoptosis, and pathogen infection. The expression levels of PoPTEN and pol-miR-novel_547, on the one hand, are regulated by pathogens and determine the outcome of pathogen infection, and, on the other hand, have a significant influence on the activation of autophagy and apoptosis, which in turn would probably affect the clearance of the invading pathogens. These results shed light on the function and regulation fish PTEN.

## 4. Materials and Methods

### 4.1. Animals and Cell Lines

The source and maintenance of Japanese flounder (average weight of 20.5 g) were as reported previously [18]. The fish were kept at 22 °C in aerated seawater for two weeks before experimental manipulation and verified to be clinically healthy as reported previously [95]. Before tissue collection, the fish were euthanized with tricaine methanesulfonate (Sigma, St. Louis, MO, USA) as reported previously [96]. The animal protocols used in this work were approved by the Ethics Committee of Institute of Oceanology, Chinese Academy of Sciences (permit No. MB1909). For cell culture, FG-9307 cells [97] were cultured at 24 °C in L-15 medium (Thermo Scientific, Waltham, MA, USA) containing 10% fetal bovine serum (FBS) (Gibco, Grand Island, NY, USA) and 100 units/mL penicillin and 100 μg/mL streptomycin (Beyotime, Shanghai, China) as reported previously [18]. HEK293T cells (CBTCCCAS, Shanghai, China) were cultured in DMEM medium (Invitrogen, Carlsbad, CA, USA) supplemented with 10% FBS, 100 units/mL penicillin, and 100 μg/mL streptomycin at 37 °C under the condition of 5% CO_2_ [18].

### 4.2. In Vivo Infection and Quantitative Real-Time PCR (qRT-PCR) Analysis of Gene Expression

Infection of flounder with *E. tarda* was performed as reported previously [18]. Briefly, *E. tarda* was cultured in LB medium at 28 °C to an OD_600_ of 0.8, the cells were washed with PBS and resuspended in PBS to 1 × 10^8^ CFU/mL. Flounder (as above) were divided randomly into two groups and injected intraperitoneally (i.p.) with 100 μL *E. tarda* or PBS. At 12 h, 24 h, and 48 h post infection (hpi), the kidney was taken aseptically from the fish (three fish at each time point). For viral infection, flounder were injected intraperitoneally (i.p.) with megalocytivirus RBIV-C1 (10^5^ copies/fish) as described previously [15]. At 2 d, 4 d, 6 d, 8 d, and 10 d post-viral infection (dpi), the kidney was taken as above. To examine the expression of pol-miR-novel_547, miRNA was extracted from the kidney tissue and reverse-transcribed using specific stem-loop primer (5′- GTCGTATCCAGTGCAGGGTCCGAGGTATTCGCACTGGATACGACAGTGAT-3′). Pol-miR-novel_547 expression was determined by qRT-PCR as reported previously using comparative threshold cycle method (2^−ΔΔCT^) with primers pol-miR-novel_547-F and pol-miR-novel_547-R (Supplementary Table S1). 5S rRNA was used as internal reference [98]. To examine PoPTEN expression during *E. tarda* and megalocytivirus infection, total RNA was extracted from the tissue with TRIzol reagent (Invitrogen, Carlsbad, CA, USA), and cDNA was synthesized with First Strand cDNA Synthesis Kit (ToYoBo, Osaka, Japan). qRT-PCR was carried out to determine PoPTEN expression as described above with primers PoPTEN-F and PoPTEN-R (Supplementary Table S1). 18S rRNA and elongation factor-1-α (EF1A) were used as internal references for *E. tarda*- and megalocytivirus-infected samples, respectively [99,100]. To examine the expression of PoPTEN in healthy flounder tissues, spleen, gill, brain, kidney, blood, intestine, muscle, heart, and liver were taken aseptically from flounder, and qRT-PCR was carried out to determine PoPTEN expression as described above.

### 4.3. RNA Mimic and Small Interfering RNA (siRNA)

The mimics of pol-miR-novel_547 and pol-miR-novel_547-M, a pol-miR-novel_547 mutant with the seed sequence (5′-CUACAGC-3′) mutated to 5′-GAUGUCG-3′, and their negative control pol-miR-NC were synthesized by GenePharma (Shanghai, China). The inhibitor of pol-miR-novel_547 and its negative control pol-miR inhibitor NC were designed and synthesized by the same company. The PoPTEN specific siRNA, siRNA-PoPTEN, and its negative control, siRNA-NC, were designed and synthesized by Ribobio (Guangzhou, China).

### 4.4. Plasmid Construction

To construct the plasmid pPTEN-Report for luciferase reporter assay, the 3′-UTR of PoPTEN was amplified by PCR using the primer pair of 3′UTR-PTEN-F/3′UTR-PTEN-R (Supplementary Table S1). The PCR product was ligated into the luciferase reporter vector pmirGLO (Promega, Fitchburg, WI, USA) at the Nhe I /Sal I enzyme sites. To construct the plasmid pPoPTEN for the overexpression of PoPTEN, the coding sequence of PoPTEN was amplified by PCR using the primer pair of CDS-PTEN-F/CDS-PTEN-R (Supplementary Table S1). The PCR product was ligated into pCN3 [101] at the EcoR V site, resulting in pPoPTEN. The plasmid pPoPTENsi used for the knockdown of PoPTEN was constructed by using siRNA as reported previously [102]. Briefly, a PoPTEN specific siRNA (5′-GCACTTCAACATCCGTCAC-3′) was inserted into the siRNA expression vector pRNAT-CMV3.1 (GenScript, Piscataway, NJ, USA) at BamH I/Alf II restriction sites, resulting in plasmid pPoPTENsi. The inhibitory effect of pPoPTENsi on PoPTEN expression in flounder tissues was verified by qRT-PCR as reported previously [102]. The negative control plasmid, pPoPTENsiC, which expresses a scramble siRNA (5′-ACCTACTGCGCTTAACACC-3′), was similarly constructed. All plasmids were verified by DNA sequencing.

### 4.5. Regulation of PoPTEN by Pol-miR-Novel-547

To determine the interaction between pol-miR-novel_547 and 3′-UTR of PoPTEN, the dual-luciferase reporter assay was performed [103]. Briefly, HEK293T cells were transfected with pPTEN-Report (control), or pPTEN-Report plus pol-miR-novel_547 mimic, pPTEN-Report plus pol-miR-NC, or pPTEN-Report plus pol-miR-novel_547 mimic-M. The transfection was carried out for 24 h using Lipofectamine 3000™ reagent (Invitrogen, Carlsbad, CA, USA) as reported previously [19]. The firefly luciferase and Renilla luciferase activities in the cells were determined using the Dual-Luciferase Reporter Assay System (Promega, Fitchburg, WI, USA). The Renilla luciferase was used as a control reporter for normalization. To determine the effect of pol-miR-novel_547 on PoPTEN expression, FG-9307 cells were transfected with or without pol-miR-novel_547 mimic or pol-miR-NC for 24 h as above. qRT-PCR was performed to determine PoPTEN expression as described above. PoPTEN protein was detected by Western blot as reported previously [104]. Briefly, the cells were lysed on ice for 30 min with RIPA lysis buffer (Beyotime, Shanghai, China). The cell lysates were mixed with 5×sodium dodecyl sulfate polyacrylamide gel electrophoresis (SDS-PAGE) loading buffer and boiled for 10 min. The samples were then subjected to 12% SDS-PAGE. Subsequently, the proteins were transferred onto a nitrocellulose blotting membrane (GE healthcare, Freiburg, Germany). The membrane was blocked in Tris-buffered saline containing Tween-20 (TBST) containing 5% skim milk at room temperature for 2 h, followed by incubation with anti-PTEN antibody (Cell Signaling Technology, Beverly, MA, USA) (1:1000 dilution), or anti-β-actin antibody (ABclonal, Wuhan, China) (1:1000 dilution) at 4 °C for overnight. The membrane was washed 3 times with TBST and incubated with HRP-conjugated anti-rabbit antibody (Abcam, Cambridge, UK) (1:2000 dilution) at room temperature for 1 h. After washing 3 times with TBST, the membrane was incubated with enhanced chemiluminescence (ECL) solution (Beyotime, Shanghai, China) and visualized using a GelDoc XR System (Bio-Rad, Hercules, CA, USA).

### 4.6. Sequence Analysis

The amino acid sequence of PoPTEN (GenBank accession number XP_019951077.1) was analyzed using the Basic Local Alignment Search Tool (BLAST) at the National Center for Biotechnology Information website (https://blast.ncbi.nlm.nih.gov/Blast.cgi) and the Expert Protein Analysis System (https://web.expasy.org/protparam/). Multiple sequence alignment was performed with DNAMAN.

### 4.7. Effect of Pol-miR-novel_547 and PoPTEN on E. Tarda Infection in FG-9307 Cells

To examine the effect of pol-miR-novel_547 on *E. tarda* infection in flounder cells, FG-9307 cells were transfected with or without pol-miR-novel_547 mimic or pol-miR-NC for 24 h as above. The transfected cells were then infected with *E. tarda* at a MOI of 20. At 2 h, 4 h, and 8 h post infection, the intracellular number of *E. tarda* was determined by plate count [19]. To examine the effect of PoPTEN on *E. tarda* infection, FG-9307 cells were transfected as above with or without siRNA-PoPTEN or siRNA-NC for 24 h. The efficiency of PoPTEN knockdown was determined by qRT-PCR as above. The transfected cells were then infected with *E. tarda*, and intracellular bacterial number was determined at 2, 4, and 8 hpi as described above.

### 4.8. Effect of PoPTEN on Bacterial and Viral Infection in Flounder

To examine the effect of PoPTEN overexpression on *E. tarda* infection in flounder, flounder were injected intramuscularly (i.m.) with pPoPTEN (1 μg plasmid/1 g fish), pCN3 (1 μg plasmid/1 g fish), or PBS (control). The kidney, spleen, and liver of the fish were harvested at 3 d, 5 d, and 7 d post-plasmid administration. The expression of PoPTEN in flounder tissues was determined by qRT-PCR as above, which showed that at 7 d post-plasmid administration, PoPTEN expression levels in the tissues were significantly increased (Supplementary Figure S4A). Therefore, 7 d post-plasmid administration was chosen for subsequent infection study as follows. The fish were administered with or without (control) pPoPTEN or pCN3 for 7 days and then infected with *E. tarda* as above. Kidney, spleen, and liver were collected aseptically at 12, 24, and 48 hpi, and bacterial recovery in the tissues was determined as reported previously [105]. To examine the effect of PoPTEN knockdown on *E. tarda* infection in flounder, flounder were injected i.m. with pPoPTENsi (1 μg plasmid/1 g fish), pPoPTENsiC (1 μg plasmid/1 g fish), or PBS (control). At 3 d, 5 d, and 7 d post-plasmid administration, kidney, spleen, and liver were harvested from the treated fish. The expression of PoPTEN in flounder tissues was determined by qRT-PCR as above. The results showed that at 7 d post-plasmid administration, PoPTEN expression levels in the tissues were significantly decreased (Supplementary Figure S4B). Therefore, 7 d post-plasmid administration was chosen for subsequent infection study as follows. The fish were administered with or without (control) pPoPTENsi or pPoPTENsiC for 7 days and then infected with *E. tarda* as above. At 12, 24, and 48 hpi, bacterial recovery in kidney, spleen, and liver was determined as above.

To examine the effect of PoPTEN overexpression on viral infection, flounder were administered with pPoPTEN, pCN3, or PBS as above. At 7 d post-plasmid administration, flounder were injected i.p. with megalocytivirus RBIV-C1 (10^5^ copies/fish). At 2 d, 4 d, 6 d, and 8 d post infection (dpi), the viral loads in kidney were determined by absolute quantitative real time RT-PCR as reported previously [15].

### 4.9. Effect of Pol-miR-novel_547 on Autophagy

To determine the effect of pol-miR-novel_547 overexpression on autophagy, FG-9307 cells were transfected with or without pol-miR-novel_547 mimic or pol-miR-NC for 24 h as above. The expression of autophagy-associated genes, i.e., ATG5, AKT, mTOR, and beclin 1, was determined by qRT-PCR as above. The sequences of all primers are listed in Supplementary Table S1. To determine the effect of pol-miR-novel_547 knockdown on autophagy, FG-9307 cells were transfected with or without pol-miR-novel_547 inhibitor or pol-miR inhibitor NC for 24 h as above. Western blot was performed as above. The primary antibodies included anti-PTEN antibody (Cell Signaling Technology, Beverly, MA, USA) (1:1000 dilution), anti-AKT antibody (Cell Signaling Technology, Beverly, MA, USA) (1:1000 dilution), anti-p-AKT antibody (Cell Signaling Technology, Beverly, MA, USA) (1:1000 dilution), anti-beclin 1 antibody (Proteintech, Chicago, IL, USA), anti-LC3B antibody (Sigma, St. Louis, MO, USA), and anti-β-actin antibody (ABclonal, Wuhan, China) (1:1000 dilution).

### 4.10. Effect of PoPTEN on Autophagy

To determine the effect of PoPTEN knockdown on autophagy in flounder cells, FG-9307 cells were transfected with or without siRNA-PoPTEN or siRNA-NC for 24 h as above. The expression of autophagy-associated genes in flounder cells was determined by qRT-PCR as above. To determine the effect of PoPTEN knockdown on autophagy in vivo, flounder were administered with pPoPTENsi, pPoPTENsiC, or PBS (control) as described above, and the expression of autophagy-associated genes in kidney was determined by qRT-PCR as above. To determine the effect of PoPTEN overexpression on autophagy in vivo, flounder were administered with pPoPTEN, pCN3, or PBS (control), and the expression of autophagy genes in kidney was determined by qRT-PCR as above. To determine the effect of PoPTEN overexpression on autophagy in mammalian cells, HEK293T cells were transfected with or without (control) pPoPTEN or pCN3 as above for 24 h. PoPTEN, AKT, p-AKT, beclin 1, and LC3 protein were detected by Western blot as above.

### 4.11. Effect of Pol-miR-novel_547 and PoPTEN on Apoptosis

FG-9307 cells were transfected with or without (control) pol-miR-novel_547 inhibitor or pol-miR inhibitor NC as above. After 24 h, the expression of PoPTEN in the transfected cells was determined by qRT-PCR as described above. Apoptosis of the transfected cells was detected using the annexin V–fluorescein isothiocyanate (FITC)/propidium iodide (PI) apoptosis detection kit (Solarbio, Beijing, China) according to the manufacturer’s instruction. Flow cytometry was conducted using a FACS Flow Cytometer (BD Biosciences, San Jose, CA, USA) as reported previously [19]. Data analysis was performed using FlowJo software v. 7.6.1 (TreeStar Inc, San Carlos, CA, USA).

### 4.12. Statistical Analysis

All of the experiments were performed at least three times, with three technical replicates for each experiment. Data analysis was conducted using two-tailed t-test and one-way analysis of variance with GraphPad Prism 6.0 Software (GraphPad Software, La Jolla, CA, USA). *p* < 0.05 was considered to be statistically significant.

## Supplementary Materials

The following are available online at https://www.mdpi.com/1422-0067/21/20/7725/s1.
