# Supplementary material for: Phosphatase and Tensin Homolog (PTEN) of Japanese Flounder—Its Regulation by miRNA and Role in Autophagy, Apoptosis and Pathogen Infection
Source: Int J Mol Sci. 2020 Oct 19;21(20):7725. doi: 10.3390/ijms21207725 (PMC7589652; doi:10.3390/ijms21207725)
Supplement: Supplementary file 1 [file ijms-21-07725-s001.pdf]

Supplementary data

Table S1. Primers used in this study.

Primer	Sequence (5'→3') [a]
pol-miR-novel_547-F	GCGCGTCTACAGCCTCTGAT
pol-miR-novel_547-R	AGTGCAGGGTCCGAGGTATT
PoPTEN-F	TCGAGGGCGTGTACAGAAAC
PoPTEN-R	CGAAGGGGTACTGTGCAACT
3'UTR-PoPTEN-F	TGTTTAAACGAGCTCGCTAGCACTCTTTTTAAACAGGAA (Nhe I)
3'UTR-PoPTEN-R	CTTGCATGCCTGCAGGTCGACTGGTTTCTTTAAGAAAAA (Sal I)
CDS-PoPTEN-F	GGCTAGCCTCGAGATATCATGGCTGCGATTATAAAA (EcoR V)
CDS-PoPTEN-F	TCCTCCTCCTCCTCCGATATCTCACACTTTTGTGATTTG (EcoR V)
ATG5-F	AACAGCTGTGGATGGGTCTG
ATG5-R	ACAGGGCGAAACAGTCTCTG
AKT-F	AGCTGATGAAGACGGAACGG
AKT-R	GGGTCTGGAGAGGAGTCCAT
mTOR-F	TCTTCCACAATCGCCAGCTT
mTOR-R	GAGCCAGTCGTCTTTGGACA
beclin 1-F	TCAATGTGCTGGATCGGGTC
beclin 1-R	GCGCTCTCTGTGGACATCAT

Underlined nucleotides are restriction sites.

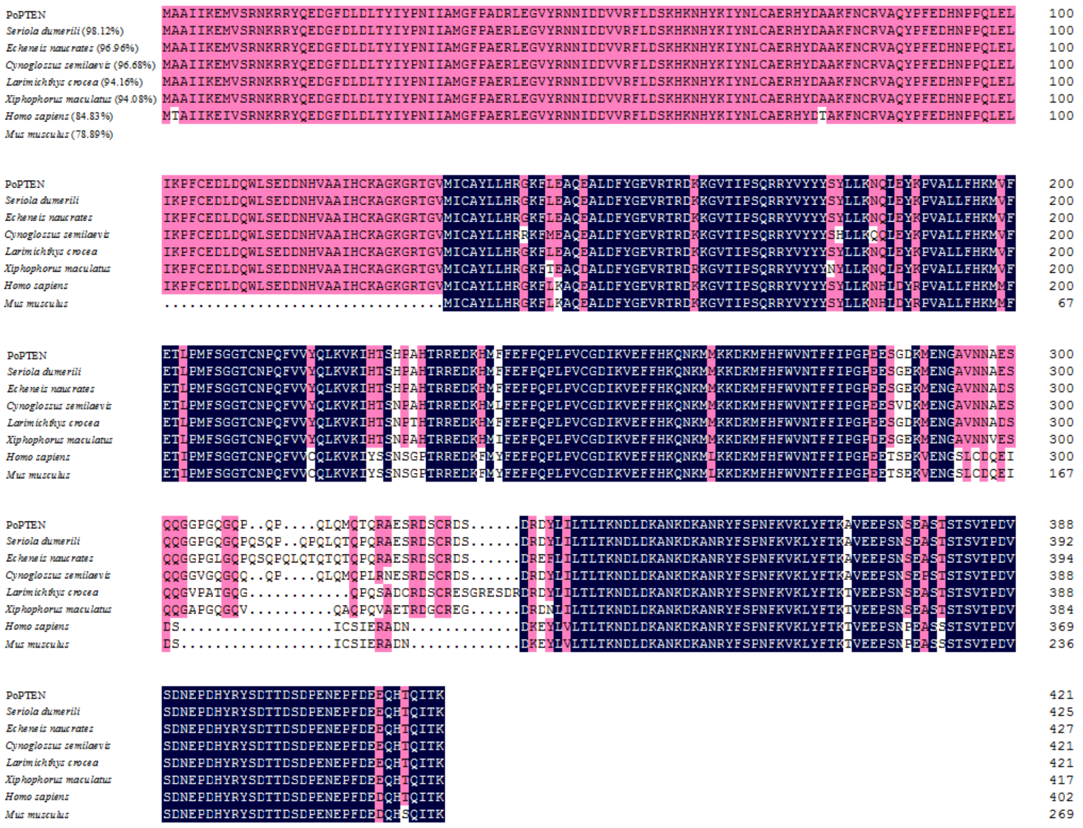

Figure S1. Alignment of the sequences of PoPTEN homologues. Dots denote gaps introduced for maximum matching. Numbers in brackets indicate overall sequence identities between PoPTEN and the compared sequences. The consensus residues are in blue, the residues that are ≥75% identical among the aligned sequences are in pink. The GenBank accession numbers of the aligned sequences are as follows:

Seriola dumerili, XP_022619472.1; *Echeneis naucrates*, XP_029377085.1; *Cynoglossus semilaevis*, XP_008320667.1; *Larimichthys crocea*, XP_010742537.1; *Xiphophorus maculatus*, XP_023183443.1; *Homo sapiens*, NP_000305.3; *Mus musculus*, XP_006526832.1.

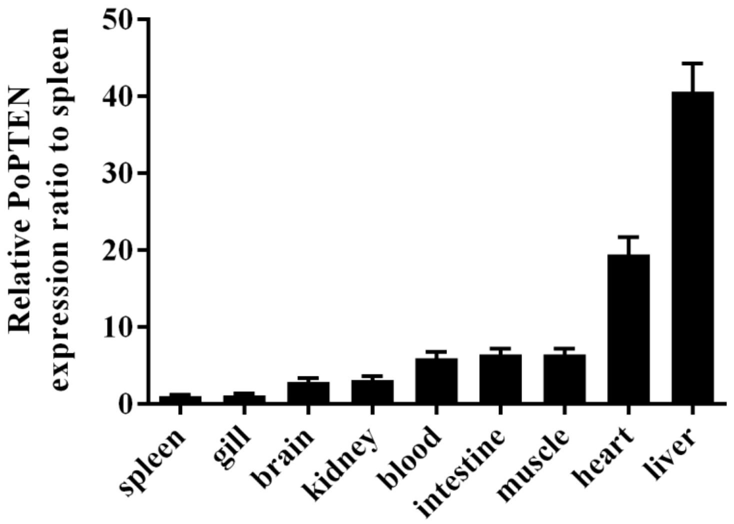

Figure S2. Tissue expression patterns of PoPTEN. PoPTEN expression in the spleen, gill, brain, kidney, blood, intestine, muscle, heart, and liver of flounder was determined by qRT-PCR. For convenience of comparison, the lowest expression level (spleen) was set as 1. Data are the means of triplicate experiments and presented as means ± SD.

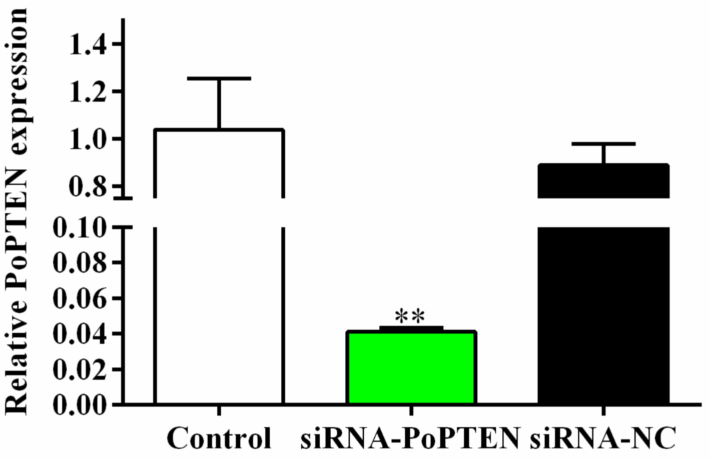

Figure S3. Verification of PoPTEN knockdown in FG-9307 cells. FG-9307 cells were transfected with or without (control) siRNA-PoPTEN, or the control siRNA (siRNA-NC). At 24 h post transfection, PoPTEN expression was measured by qRT-PCR. Values are the means of triplicate experiments and shown as means ± SEM. ** $p < 0.01$.

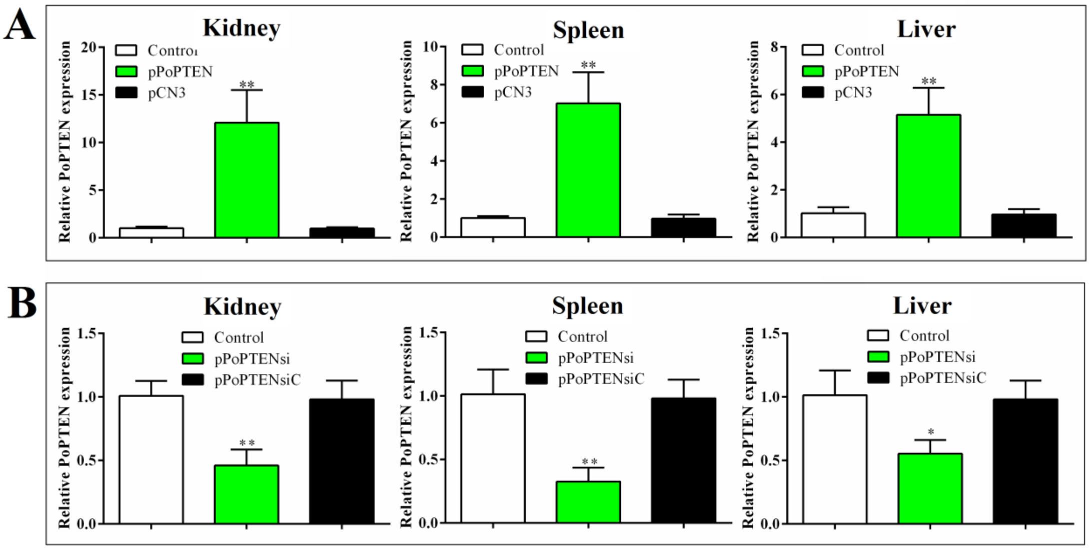

Figure S4. Verification of PoPTEN overexpresion (**A**) and knockdown (**B**) in flounder. For PoPTEN overexpression, flounder were administered with or without (control) pPoPTEN or the control plasmid pCN3 (**A**); for PoPTEN knockdown, flounder were administered with or without (control) pPoPTENsi or the control plasmid pPoPTENsiC (**B**). In both panels, PoPTEN expression in kidney, spleen, and liver was determined by qRT-PCR at 7d post-plasmid injection. In all tissues, the expression level of the control fish was set as 1. Values are the means of triplicate experiments and shown as means ± SD. ** $p < 0.01$; *$p < 0.05$.

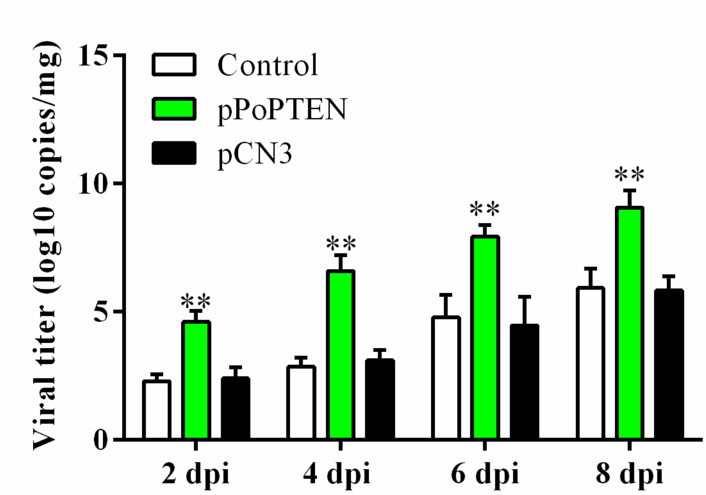

Figure S5. The effect of PoPTEN on megalocytivirus infection. Flounder were were administered with or without (control) pPoPTEN or the control plasmid pCN3 and then infected with megalocytivirus. The viral load in kidney was determined at 2, 4, 6, and 8 days post-injection (dpi). Values are shown as means ± SD ($n = 3$), **$p < 0.01$.
